# Pancréatite surinfectée révélé par un abcès du psoas

**DOI:** 10.11604/pamj.2014.19.387.4360

**Published:** 2014-12-17

**Authors:** Coulibaly Mahamadoun, Houari Nawfal, Boukatta Brahim, Hicham Sbai, Kanjaa Nabil

**Affiliations:** 1 Service de Réanimation Polyvalente A4, CHU Hassan 2 de Fès, Maroc

**Keywords:** Pancréatite, surinfection de coulées de nécrose, fistulisation, abcès, muscle psoas, Pancreatitis, superinfection of necrosis flows, fistulisation, abscess, psoas muscle

## Abstract

Les pancréatites aigues grave peuvent se compliquer de pseudo kystes qui peuvent conduire à la formation de fistules, ces fistules peuvent être dirigées vers différentes régions avec différentes manifestations cliniques. Ces manifestations extra pancréatiques de la pancréatite aigue constituent, par leurs particularités cliniques, biologiques et radiologiques, un réel apport au diagnostic positif. Le pronostic est celui de la pancréatite et dépend du site de la fistulisation. Le traitement par laparotomie a longtemps été le «Gold Standard», ces dernières années ont vu le développement de moyens moins invasifs et donc offrants un minimum de morbi-mortalité (Chirurgie mini invasive, drainage percutané). Nous rapportons l'observation d'un patient ayant séjourné dans notre structure.

## Introduction

La pancréatite aiguë est une pathologie qui reste grave dans près de 20% des cas. Cette gravité est définie par l'existence d'une défaillance multiviscérale et/ou la survenue d'une complication locale. Les complications locales émanent le plus souvent de la nécrose, d'un abcès ou d'un pseudokyste. Ces pseudokystes pancréatiques peuvent être complexes et se manifester de différentes manières (infections, saignements, compression d'organes adjacents, atteintes vasculaires et ou thrombotiques) ou se fistuliser (région retro péritonéale, le long du muscle Psoas ou au niveau de la région périnéale) [1]. Ces manifestations extra pancréatiques de la pancréatite aiguë constituent, par leurs particularités cliniques, biologiques et radiologiques, un réel apport au diagnostic positif. Le cas de fistulisation au muscle Psoas reste encore une entité rare cependant qui n'est pas à méconnaitre, nous rapportons l'observation d'un patient ayant séjourné dans notre structure.

## Patient et observation

**Figure 1:**
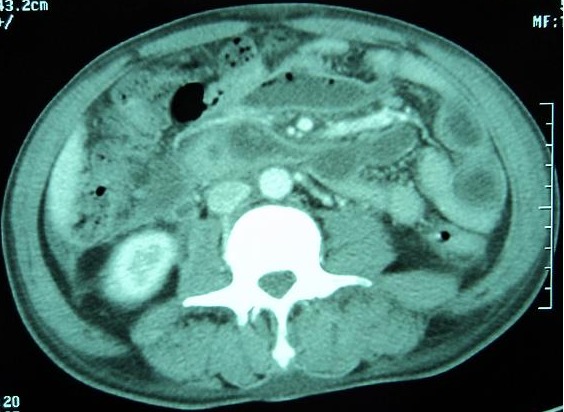
Collections intra pancréatique avec extensions au chef lombaire du muscle psoas droit

**Figure 2:**
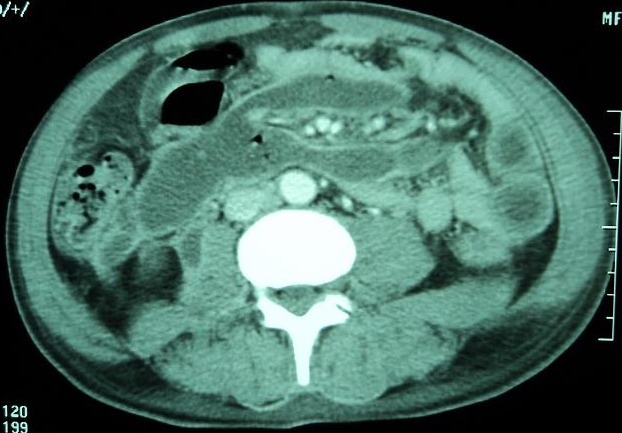
Collections avec présence de bulles d'air péripancréatique,collection du muscle Psoas

**Figure 3:**
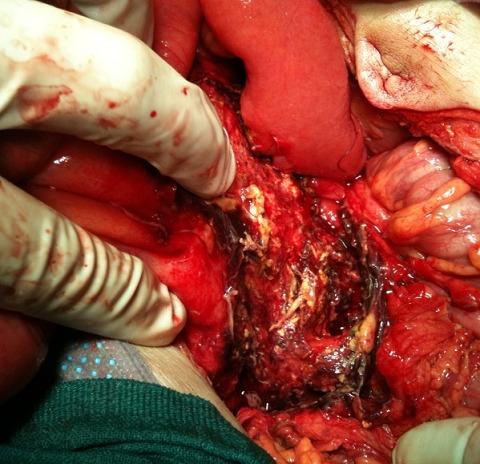
Drainage chirurgicale des coulées de nécroses peripancréatique

**Figure 4:**
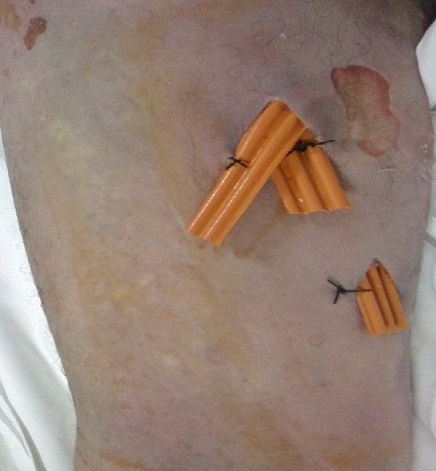
Ddrainage par lame de Delbet de l'abces du Psoas

Nous rapportons l'observation de B.L, âgé de 65 ans, diabétique sous ADO depuis 6 ans ayant consulté aux urgences pour psoïtis et impotence fonctionnelle du membre inférieur droit évoluant depuis 5 jours. L'anamnèse trouve comme antécédent récent une hospitalisation 20 jours auparavant en chirurgie viscérale pour Pancréatite aiguë stade D de Balthazar, au cours de cette première hospitalisation, il avait un taux de Lipase sérique à 34 fois la normal soit 6520 UI/l, il était apyrétique sans syndrome infectieux ni autres signes de défaillances multi viscérale, l’évolution était favorable et le patient fut déclaré sortant avec surveillance à domicile après 72 heures d'hospitalisation. L'examen a son admission trouvait,un patient conscient GCS: 15; ses constantes hémodynamique étaient: PAS: 120 mm hg; PAD: 65 mm hg; tachycarde à 120 b/min; il était fébrile à 39°c;polypnéique à 25 c/min;l'examen abdominale trouvait une distension abdominale avec défense péri-ombilicale sans signes inflammatoire; le psoitis était franc et invincible avec une collection douloureuse au niveau de la face interne de la cuisse droite. Le reste de l'examen somatique trouvait des plies de déshydratations et des râles sous crépitant à l'auscultation pleuro pulmonaire. Apres mise en condition (Monitorage, prise d'une voie veineuse jugulaire interne droite, sondage gastrique et vésicale) un bilan biologique et radiologique ont été réalisé et trouvaient: Un syndrome infectieux avec une hyperleucocytose à 32100 GB/mm^3^ et une CRP à 320mg/l; la lipasémie était à 965 UI/l soit 5 fois la normale, le reste du bilan biologique était sans particularité notamment la fonction rénale, le bilan hépatique et l'ionogramme sanguin. La TDM abdominale injectée trouvait une pancréatite « E » de Bathazar avec coulées de nécroses surinfectées, communicant avec le chef lombaire du Psoas droit siège d'une collection en son sein (Figure 1, Figure 2). Sous couverture antibiotique (imipenème) le patient a été acheminé au bloc opératoire où un double abord chirurgical a été réalisé: abdominal pour drainage chirurgical des coulées de nécrose, cholécystectomie, lavage abondant et mis en place de drains (Figure 3); abord inguinale pour mise à plat et drainage de l'abcès du Psoas (Figure 4). Les prélèvements de pus per opératoire ont mis en évidence un Pseudomonas *aeruginosa*. Les suites post opératoires ont été marquées par la survenue d'un état de choc septique réfractaire, le patient est décédé dans un tableau de défaillance multiviscérale 4 (quatre) jours après la chirurgie.

## Discussion

Les travaux de Molmenti et al. [2, 3] ont montré, grâce à l'injection de latex dans la tête du pancréas de cadavres, qu'il existe un plan de communication rétromésentérique s’étendant par la face antérolatérale du psoas jusqu'au pelvis et à la racine du membre pelvien. Ainsi, une surinfection de coulées de nécrose ou un faux kyste du pancréas peuvent se prolonger dans le psoas par l'espace interaorticocave, ou latérocave, comme c'est le cas dans notre observation. De nombreux cas de fistules pancréatiques ont été décrits, essentiellement dans le tube digestif, dans les séreuses, dans la voie biliaire principale et dans les vaisseaux [4], d'autres moins fréquents ont été decrit dans la littérature (plèvre,scrotum); cependant la localisation de fistules pancréatico-psoas reste encore une entité assez rare, nous en avons trouvé 11(onze) à ce jour [1, 5–13]; la symptomatologie est géneralement une masse douloureuse de l'aine, seuls 2 patients se sont présentés avec douleur et impotence fonctionnelle de la hanche. Les fistulisations extra pancréatique surviennent en moyenne 2 à 3 semaines après l’épisode de pancréatite aiguë. Les seuils de CRP en faveur d'une surinfection varient beaucoup en fonction des études, mais une valeur de 150 mg/L est classiquement retenue [14]. Plusieurs études dont une récente méta-analyse, ont rappelé l'intérêt de la procalcitonine comme marqueur précoce du diagnostic de cette complication dont elle est fondamentalement plus spécifique que la CRP [15]. La prise en charge habituelle des fistules pancréatiques repose sur la chirurgie d'exérèse ou de dérivation cependant elle s'accompagne d'une morbi-mortalité assez lourde d'où le développement ces dernières années des techniques de drainage percutané [16], cependant les données de la littérature sur l'efficacité du drainage percutané sont très hétérogènes. Les résultats sont globalement décevants avec un taux de succès inférieur à 30% [17]. Il peut avoir un rôle temporisateur qui a été souligné pour des patients précaires en défaillance multiviscérale. Les nouvelles techniques mini-invasives sont particulièrement intéressantes lorsque la fistule siège sur le pancréas céphalo-isthmique car la chirurgie à ce niveau est plus difficile que sur le pancréas gauche, elle repose sur deux techniques: le débridement par cœlioscopie et le débridement rétropéritonéal vidéo-assisté. Cette dernière permet d’éviter une contamination péritonéale mais l'extraction des tissus nécrotiques peut être limitée, et plusieurs reprises sont souvent nécessaires. L'antibiothérapie est de règles devant des signes clinico-biologique en faveur de la surinfection des coulées de nécroses, elle vise habituellement les bacilles gram négatifs et les germes anaérobies.

## Conclusion

Les pancréatites aiguës grave se compliquent généralement de pseudokystes qui peuvent conduire à la formation de fistules, ces fistules peuvent être dirigées vers différentes régions avec différentes manifestations cliniques. Le pronostic est celui de la pancréatite et dépend du site de la fistulisation. Le traitement par laparotomie a longtemps été le «Gold Standard», ces dernières années ont vu le développement de moyens moins invasifs et donc offrants un minimum de morbi-mortalité (Chirurgie mini invasive, drainage percutané).
